# Transcriptome profiling of porcine testis tissue reveals genes related to sperm hyperactive motility

**DOI:** 10.1186/s12917-020-02373-9

**Published:** 2020-05-26

**Authors:** Maren van Son, Nina Hårdnes Tremoen, Ann Helen Gaustad, Dag Inge Våge, Teklu Tewoldebrhan Zeremichael, Frøydis Deinboll Myromslien, Eli Grindflek

**Affiliations:** 1 Norsvin SA, 2317 Hamar, Norway; 2 Department of Biotechnology, Inland Norway University of Applied Sciences, 2318 Hamar, Norway; 3 Centre for Integrative Genetics (CIGENE), Department of Animal and Aquacultural Sciences, Faculty of Biosciences, Norwegian University of Life Sciences, 1432 Ås, Norway

**Keywords:** Sperm hyperactivity, Hyperactive motility, Testis, Gene expression, Pig, RNA sequencing

## Abstract

**Background:**

Sperm hyperactive motility has previously been shown to influence litter size in pigs, but little is known about the underlying biological mechanisms. The aim of this study was to use RNA sequencing to investigate gene expression differences in testis tissue from Landrace and Duroc boars with high and low levels of sperm hyperactive motility. Boars with divergent phenotypes were selected based on their sperm hyperactivity values at the day of ejaculation (day 0) (contrasts (i) and (ii) for Landrace and Duroc, respectively) and on their change in hyperactivity between day 0 and after 96 h liquid storage at 18 °C (contrast (iii)).

**Results:**

RNA sequencing was used to measure gene expression in testis. In Landrace boars, 3219 genes were differentially expressed for contrast (i), whereas 102 genes were differentially expressed for contrast (iii). Forty-one differentially expressed genes were identified in both contrasts, suggesting a functional role of these genes in hyperactivity regardless of storage. Zinc finger *DNLZ* was the most up-regulated gene in contrasts (i) and (iii), whereas the most significant differentially expressed gene for the two contrasts were ADP ribosylation factor *ARFGAP1* and solute carrier *SLC40A1*, respectively. For Duroc (contrast (ii)), the clustering of boars based on their gene expression data did not reflect their difference in sperm hyperactivity phenotypes. No results were therefore obtained for this breed. A case-control analysis of variants identified in the Landrace RNA sequencing data showed that SNPs in *NEU3*, *CHRDL2* and *HMCN1* might be important for sperm hyperactivity.

**Conclusions:**

Differentially expressed genes were identified in Landrace boars with high and low levels of sperm hyperactivity at the day of ejaculate collection and high and low change in hyperactivity after 96 h of sperm storage. The results point towards important candidate genes, biochemical pathways and sequence variants underlying sperm hyperactivity in pigs.

## Background

The use of artificial insemination (AI) is crucial for efficient pig production and proper evaluation of semen quality is essential for high quality AI. Microscopic evaluation of ejaculates is a commonly used method but is subjective and not able to distinguish between subpopulations of spermatozoa with different motility characteristics [1]. Computer-Assisted Semen Analysis (CASA) provides accurate and objective evaluations of many sperm characteristics such as motility, morphology and concentration [1], and has been used in several species to predict fertility outcome [2]. Ejaculated mammalian spermatozoa are not able to fertilize an oocyte before capacitation has occurred, a process where they acquire hyperactive motility and other modifications that facilitate fertilization [3]. The hyperactive motility is characterized by a vigorous and non-linear swimming pattern, which help the spermatozoa to penetrate the zona pellucida [4]. CASA recognizes the hyperactive motility pattern as high curvilinear velocity and amplitude of the lateral head movement and low linear trajectory [5]. Hyperactive swimming pattern varies between species, and for boar spermatozoa the CASA thresholds have been related to curvilinear velocity (VCL), lateral head displacement (ALH), linearity (LIN) and wobble (WOB) [5]. The change in swimming pattern towards hyperactivity has been shown to require calcium, increased pH and ATP production [4].

In our previous study, several parameters defining hyperactive motility was correlated to the total number of piglets born, and breed differences were found for sperm motility characteristics [6]. Whereas Landrace developed more hyperactivity during storage, Duroc showed a large portion of sperm cells with hyperactive swimming pattern already at the day of ejaculate collection and no significant increase after storage [6]. Due to factors such as long-distance shipment, the semen doses are often stored for 48 to 96 h before used for insemination. Differences in hyperactive motility between the day of semen collection and after storage is of interest. The sperm cells’ capability of obtaining and maintaining hyperactive motility is a prerequisite for fertilization and if this occurs too early, there is a risk that the sperm cells deplete their energy store and die before they reach the oocyte [3].

Previous studies on the genomics underlying sperm hyperactive motility have focused on a limited number of candidate genes. A family of CatSper (cation channel of sperm) genes has been associated with different sperm quality parameters, including hyperactive motility [reviewed by 7]. CatSpers are voltage-dependent, calcium ion selective and pH-sensitive ion channels that controls entry of calcium ions into sperm cells [7, 8]. Other candidate genes have been associated to sperm hyperactivation in other species, like a family of β-defensin genes in mouse [9]. A proteomic study of boar spermatozoa have identified several proteins important for capacitation, belonging to pathways such as tricarboxylic acid cycle, glutathione metabolism, adipocytokine signaling and insulin action [10].

Before the spermatozoa can acquire hyperactive motility and fertilize an oocyte, it must undergo several different processes within testes including production, maturation and ejaculation. In this study, we wanted to examine how gene expressions in testis are associated with hyperactivity in ejaculated spermatozoa, as production and maturation of spermatozoa takes place in testis. Testis tissue samples were therefore collected from Landrace and Duroc boars where spermatozoa had been examined for degree of hyperactivity at the day of ejaculation (day 0) and after 96 h storage. Samples with high and low sperm hyperactive motility at day 0 and high and low change in level of hyperactivity between day 0 and 96 h storage were then used for transcriptome sequencing and facilitated the identification of differentially expressed genes and pathways.

## Results

### Sperm hyperactive motility data

Transcriptome sequencing was performed to analyze gene expression in testis tissue from Landrace and Duroc boars with different levels of sperm hyperactive motility. Three different contrasts were prepared in this study: (i) high versus low hyperactivity at ejaculation (day 0) in Landrace, (ii) high versus low hyperactivity at ejaculation (day 0) in Duroc, and (iii) high versus low change in levels of hyperactivity between day 0 and 96 h storage in Landrace (Table 1). In Duroc, a large proportion of the sperm cells were already hyperactive at day 0, and there were not enough extreme animals to make a contrast after 96 h storage. The mean (±SD) hyperactivity values for the contrasts were 14.5% (±1.7) (*n* = 4) and 1.6% (±0.3) (*n* = 4) for contrast (i), 26.7% (±1.7) (*n* = 5) and 6.3% (±2.7) (*n* = 4) for contrast (ii), and 15.0% (±3.0) (*n* = 3) and 2.7% (±0.6) (*n* = 4) for contrast (iii), respectively.

**Table 1:** Sperm hyperactivity measurements for the different boars included in this study

Group	Boar	n (ejaculates)	mean %hyperactivity	SD %hyperactivity	Read depth
Landrace low day 0	L1	3	1.9	0.2	77.6
	L2	4	1.2	0.7	107.7
	L3	3	1.6	0.6	119.9
	L4	3	1.6	0.2	87.7
Landrace high day 0	L5	3	16.4	7.3	94.6
	L6	3	13.6	3.2	113.6
	L7	4	15.5	3.1	127.4
	L8	4	12.6	3.1	139.7
Landrace low change 96 h vs. day 0	L9	5	3.3	2.1	80.0
	L10	3	2.1	1.4	78.9
	L11	4	2.3	0.6	107.7
	L12	3	3.0	2.8	71.2
Landrace high change 96 h vs. day 0	L13	3	11.7	7.0	121.4
	L14	4	17.3	9.0	127.4
	L15	3	16.2	3.7	84.6
Duroc low day 0	D1	3	3.7	0.7	68.5
	D2	3	7.0	2.8	72.0
	D3	4	9.8	2.4	77.8
	D4	3	4.7	1.5	86.7
Duroc high day 0	D5	3	23.6	7.9	65.2
	D6	4	23.8	3.9	74.6
	D7	3	27.9	4.0	76.2
	D8	3	27.4	1.4	121.2
	D9	3	27.9	8.7	195.7

The boars of contrast (i) (L1-L8), (iii) (L9-L15) and (ii) (D1-D9) are presented with the number of ejaculates, mean and standard deviation (SD) for %hyperactivity in the ejaculates. Read depth is number of sequenced reads per sample in million

### Testis transcriptome sequence data

The average number of 100 bp single reads generated by RNA sequencing across the samples was 98 million, ranging from 65 to 196 million (Table 1). Trimmomatic [11] removed between 0.38–0.7% of the reads, and 96.44% of the remaining high-quality reads were successfully mapped to the pig genome build 11.1 [12]. For the different contrasts, 82–83% of the 25,880 pig genes had at least one count in at least one of the samples and were included in the differential expression analysis. Normalization in edgeR adjusted for differences in sequencing depths as represented by differing library sizes.

### Differential expression

**Fig. 1:**
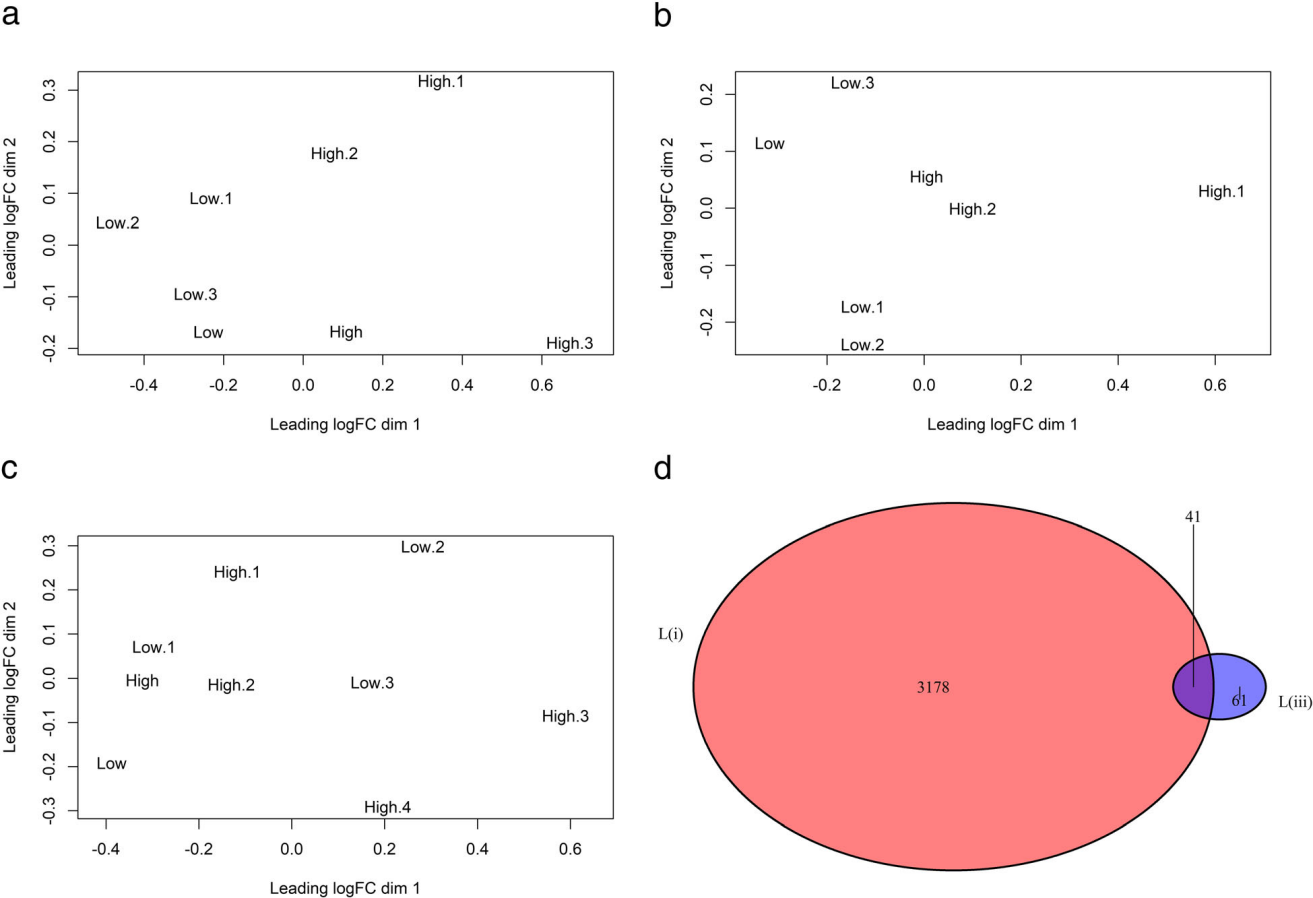
Multidimensional scaling (MDS) plots and Venn diagram for the gene expression data. **a** and **b** are MDS plots of contrasts (i) and (iii) in Landrace, respectively, whereas **c** is of contrast (ii) in Duroc. **d** shows the number of differentially expressed genes for contrast (i) and (iii) with their overlap

**Fig. 2:**
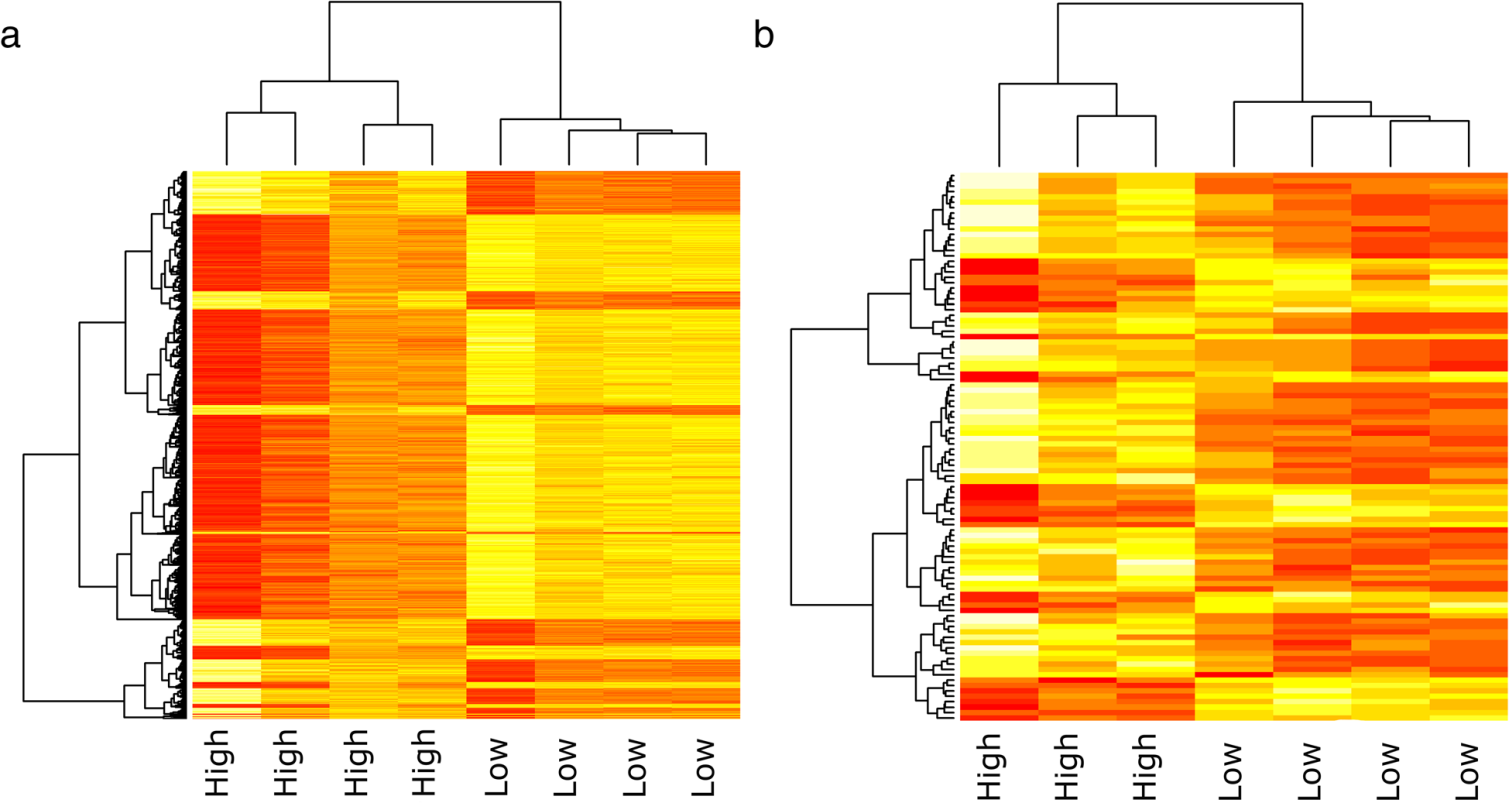
Heatmap for hierarchical clustering of differential gene expression. Each row of the grid corresponds to a different gene and each column represents a sample. The up-regulated (red) and down-regulated (yellow) genes were distinguished between the high versus low sperm hyperactivity groups in **a**) contrast (i) high versus low hyperactivity at the day of ejaculation in Landrace and **b**) contrast (iii) high versus low change in levels hyperactivity between day 0 and 96 h storage in Landrace

Contrasts (i) and (iii) showed similarity in gene expression patterns when performing cluster analysis by multidimensional scaling (Fig. 1 A and B). Differentially expressed genes are displayed in heat maps (Fig. 2 A and B), showing similar trends for the high and low groups. Contrast (ii), on the other hand, did not cluster as expected based on sperm hyperactivity measurements as described above (Fig. 1C). No significant results could therefore be obtained from the Duroc contrast (ii).

For contrast (i), high versus low hyperactivity on day 0 in Landrace, 3219 genes were found differentially expressed (FDR < 0.05) [see Additional file 1]. Of these, 870 genes were down-regulated in the high hyperactivity group whereas the rest were up-regulated. The most significant differentially expressed gene was ADP ribosylation factor GTPase activating protein 1 (*ARFGAP1*) (FDR = 1.2e-05). The most up-regulated gene in the high hyperactivity group was DNL-type zinc finger (*DNLZ*) (logFC = 2.7), whereas the most down-regulated gene was novel gene *ENSSSCG00000037477* (NCBI description: tetratricopeptide repeat protein 37-like; logFC = − 3.0).

For contrast (iii), high versus low change in levels of hyperactivity between day 0 and 96 h storage in Landrace, 102 genes were differentially expressed (FDR < 0.05) [see Additional file 2]. Of these, 33 genes were up-regulated in the high hyperactivity group whereas 69 were down-regulated, and the most significant gene was solute carrier family 40 member 1 (*SLC40A1*) (FDR = 9.7e-04). In the high hyperactivity group, the most up-regulated gene was the same as in contrast (i): *DNLZ* (logFC = 1.9), whereas the most down-regulated gene was calcium dependent secretion activator (*CADPS*) (logFC = − 2.0).

**Fig. 3:**
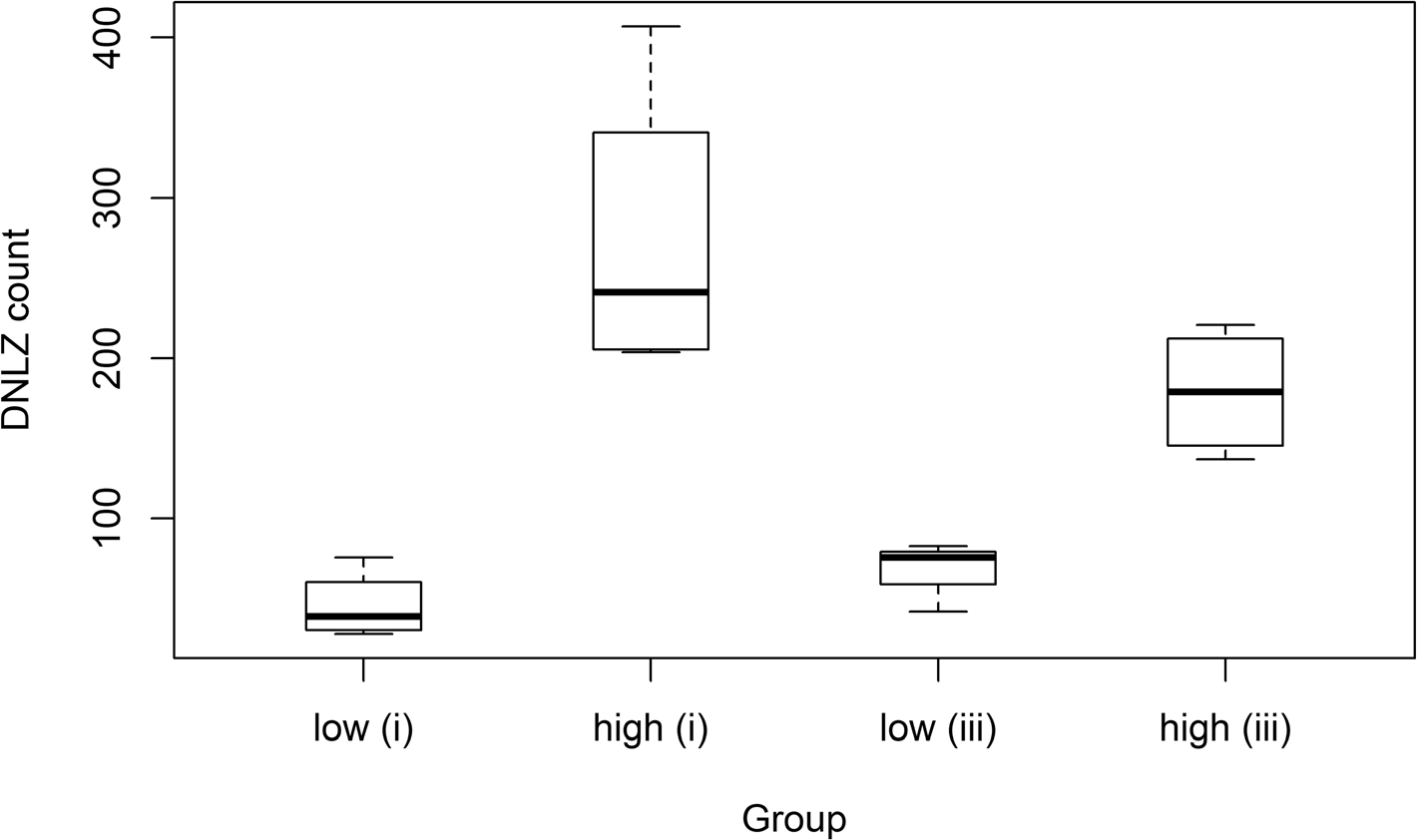
Boxplot showing the differential expression of *DNLZ*. The most significant gene in common for the Landrace contrasts (i) high versus low hyperactivity at the day of ejaculation in Landrace and (iii) high versus low change in levels hyperactivity between day 0 and 96 h storage in Landrace was *DNLZ*. The read counts for the high and low groups of animals in these two contrasts are plotted and box edges represents the upper and lower quartile with the median value shown as a bold line in the middle of the box. Whiskers represent 1.5 times the quartile of the data

Between contrasts (i) and (iii), 41 differentially expressed genes were found in common (Fig. 1D and Table 2). The most significant common gene was *DNLZ* (Fig. 3).

**Table 2:** Differentially expressed genes in common for contrasts (i) high versus low hyperactivity at day 0 in Landrace and (iii) high versus low change in levels of hyperactivity between day 0 and 96 h storage in Landrace

Gene ID	Gene symbol	Gene name	FDR (i)	FDR (iii)	Count high (i)	Count low (i)	Count high (iii)	Count low (iii)
*ENSSSCG00000034732*	*DNLZ*	DNL-type zinc finger	4.5e-08	1.3e-03	182 (± 22)	1093 (± 95)	67 (± 22)	179 (± 40)
*ENSSSCG00000035243*	*RAB27B*	RAB27B, member RAS oncogene family	2.1e-02	7.7e-03	1650 (± 1105)	710 (± 259)	1706 (± 801)	519 (± 226)
*ENSSSCG00000012405*	*RPS4X*	ribosomal protein S4, X-linked	3.0e-02	1.0e-02	31,095 (± 13,846)	18,108 (± 5267)	34,635 (± 13,478)	14,745 (± 5991)
*ENSSSCG00000008857*	*MSMO1*	methylsterol monooxygenase 1	2.3e-02	1.0e-02	11,766 (± 7534)	5367 (± 1735)	11,744 (± 2348)	4422 (± 2427)
*ENSSSCG00000030005*	*LGALSL*	galectin like	2.9e-02	1.5e-02	3051 (± 1446)	1646 (± 488)	3916 (± 1783)	1433 (± 505)
*ENSSSCG00000014139*	*ARSK*	arylsulfatase family member	1.1e-02	1.5e-02	913 (± 303)	466 (± 108)	992 (± 131)	364 (± 110)
*ENSSSCG00000034499*	*TMEM225B*	transmembrane protein 225B	1.7e-03	1.5e-02	895 (± 330)	2094 (± 786)	933 (± 187)	1479 (± 577)
*ENSSSCG00000003575*	*CD164L2*	CD164 molecule like 2	7.3e-03	1.5e-02	109 (± 73)	361 (± 182)	103 (± 18)	241 (± 63)
*ENSSSCG00000014156*	*ARRDC3*	arrestin domain containing	1.7e-03	2.2e-02	3734 (± 1606)	1730 (± 426)	4836 (± 2569)	1659 (± 352)
*ENSSSCG00000000602*	*RERG*	RAS like estrogen regulated growth inhibitor	8.2e-04	2.2e-02	848 (± 607)	241 (± 65)	854 (± 625)	198 (± 97)
*ENSSSCG00000032235*	*ARFGAP1*	ADP ribosylation factor GTPase activating protein	1.2e-05	2.3e-02	322 (± 96)	1043 (± 387)	339 (± 84)	686 (± 240)
*ENSSSCG00000016027*	*ITGAV*	integrin alpha-V precursor	4.9e-02	2.4e-02	7399 (± 3982)	4034 (± 1544)	8956 (± 4183)	3467 (± 1892)
*ENSSSCG00000031571*	novel gene	novel gene	4.3e-05	2.4e-02	553 (± 68)	1395 (± 316)	343 (± 181)	726 (± 375)
*ENSSSCG00000027178*	*MAN2B2*	mannosidase alpha class 2B	5.1e-05	2.5e-02	161 (± 49)	489 (± 150)	166 (± 78)	304 (± 94)
*ENSSSCG00000005671*	*CRAT*	carnitine O-acetyltransferase	2.6e-04	2.7e-02	3346 (± 1334)	7076 (± 1745)	3539 (± 1249)	4976 (± 849)
*ENSSSCG00000027472*	*GCA*	grancalcin	9.9e-03	2.8e-02	3324 (± 1663)	1575 (± 516)	3584 (± 1860)	1268 (± 665)
*ENSSSCG00000010084*	novel gene	novel gene	0.0002	2.8e-02	1785 (± 534)	3575 (± 743)	1628 (± 671)	2559 (± 935)
*ENSSSCG00000028425*	*UBL4B*	ubiquitin like 4B	1.4e-05	2.8e-02	1450 (± 700)	4446 (± 1009)	1469 (± 746)	2832 (± 1106)
*ENSSSCG00000008118*	*PROM2*	prominin 2	2.0e-02	2.8e-02	76 (± 54)	210 (± 77)	74 (± 21)	200 (± 82)
*ENSSSCG00000033277*	*IQCF5*	IQ motif containing F5	8.1e-03	2.9e-02	18,391 (± 3501)	26,423 (± 2197)	16,727 (± 4257)	20,458 (± 4997)
*ENSSSCG00000007700*	*HIP1*	huntingtin interacting protein 1	8.7e-04	3.7e-02	3592 (± 993)	5921 (± 1011)	2947 (± 1720)	4441 (± 1402)
*ENSSSCG00000035218*	*ADA2*	adenosine deaminase CECR1 precursor	4.2e-02	3.8e-02	182 (± 47)	303 (± 68)	137 (± 36)	243 (± 27)
*ENSSSCG00000013335*	*LGR4*	leucine rich repeat containing G protein-coupled	3.8e-02	4.0e-02	3815 (± 1202)	2412 (± 767)	5078 (± 2454)	2098 (± 623)
*ENSSSCG00000007019*	*GPAT4*	glycerol-3-phosphate acyltransferase 4	1.5e-04	4.0e-02	6146 (± 1491)	10,211 (± 2337)	5818 (± 2115)	7630 (± 2240)
*ENSSSCG00000016592*	*FSCN3*	fascin-3	5.9e-04	4.0e-02	7738 (± 2188)	13,775 (± 2808)	6367 (± 3262)	10,079 (± 3560)
*ENSSSCG00000016278*	*TEX44*	testis expressed 44	2.1e-04	4.4e-02	4149 (± 2414)	11,049 (± 2567)	4330 (± 2331)	7558 (± 3339)
*ENSSSCG00000006928*	*LMO4*	LIM domain only 4	3.3e-02	4.8e-02	831 (± 261)	465 (± 121)	989 (± 385)	368 (± 156)
*ENSSSCG00000032715*	*CERS6*	ceramide synthase 6	3.6e-02	4.8e-02	4029 (± 1640)	2452 (± 690)	6135 (± 3651)	2277 (± 572)
*ENSSSCG00000033734*	*TMSB4Y*	thymosin beta 4, Y-linked	1.8e-02	4.8e-02	3676 (± 1035)	2191 (± 602)	5172 (± 3085)	1951 (± 352)
*ENSSSCG00000028613*	*CETN1*	centrin 1	3.1e-04	4.8e-02	31,883 (± 6281)	54,982 (± 9504)	30,604 (± 7660)	38,067 (± 8989)
*ENSSSCG00000003949*	*CDC20*	Cell division cycle protein 20 homolog	6.8e-04	4.8e-02	3008 (± 1098)	5940 (± 1740)	3005 (± 1079)	4302 (± 1296)
*ENSSSCG00000021337*	*PRM1*	protamine 1	8.2e-03	4.8e-02	82,199 (± 27,212)	117,697 (± 11,624)	67,279 (± 46,538)	100,121 (± 16,807)
*ENSSSCG00000029145*	*POMGNT1*	protein O-linked-mannose beta-1,2-N-acetylglucosaminyltransferase 1	1.5e-02	4.8e-02	2003 (± 748)	3583 (± 732)	1930 (± 356)	3052 (± 895)
*ENSSSCG00000039792*	*CDRT4*	CMT1A duplicated region transcript 4	1.7e-03	4.8e-02	466 (± 116)	923 (± 210)	428 (± 121)	719 (± 219)
*ENSSSCG00000035607*	*PRM3*	protamine 3	8.9e-05	4.8e-02	2567 (± 1570)	6555 (± 1223)	2693 (± 1803)	4481 (± 1585)
*ENSSSCG00000003486*	*ARHGEF10L*	Rho guanine nucleotide exchange factor 10 like	1.1e-03	4.8e-02	134 (± 53)	356 (± 180)	140 (± 58)	279 (± 91)
*ENSSSCG00000013519*	*ZNRF4*	zinc and ring finger 4	2.4e-05	4.8e-02	1019 (± 587)	3079 (± 966)	1112 (± 647)	2049 (± 805)
*ENSSSCG00000014187*	*NUDT12*	nudix hydrolase 12	2.6e-02	4.9e-02	792 (± 307)	419 (± 131)	928 (± 418)	340 (± 136)
*ENSSSCG00000002839*	*C16orf78*	chromosome 16 open reading frame 78	1.8e-03	4.9e-02	3755 (± 1189)	6066 (± 1030)	3256 (± 1979)	4673 (± 1694)
*ENSSSCG00000021448*	*CCDC17*	coiled-coil domain containing 17	5.9e-06	4.9e-02	1719 (± 517)	3650 (± 792)	1689 (± 730)	2647 (± 1024)
*ENSSSCG00000021255*	*ADA*	adenosine deaminase	1.7e-02	4.9e-02	82 (± 23)	162 (± 37)	63 (± 34)	152 (± 65)

Genes differentially expressed in contrasts (i) and (iii) are presented with gene Ensembl ID, gene symbol, gene name and significance level (FDR) in Landrace (L) for the two contrasts. Moreover, average read counts for the high and low groups are included along with standard deviations

### Gene ontology

Over-represented pathways among the differentially expressed genes were identified by gene ontology analysis. For contrast (i), 12 pathways were over-represented (Table 3), and the most significant pathways include the “extracellular exosome” (GO:0070062), “cytoplasm” (GO:0005737) and “in utero embryonic development” (GO:0001701). Furthermore, all genes belonging to the biological process pathway “establishment or maintenance of transmembrane electrochemical gradient” (GO:0010248) were differentially expressed: ATPase gene members *ATP1A1*, *ATP1A2*, *ATP1B2*, *ATP4A* and BCL2 associated X, apoptosis regulator (*BAX*). Eight of the nine genes in the cellular component “GATOR2 complex” (GO:0061700) were also differentially expressed: cytosolic arginine sensor for mTORC1 subunit 1 (*CASTOR1*), meiosis regulator for oocyte development (*MIOS*), SEC13 homolog nuclear pore and COPII coat complex component (*SEC13*), sestrins *SESN1* and *SESN2*, KICSTOR complex subunit (*SZT2*), and WD (tryptophan-aspartic acid dipeptide) repeat domains *WDR24* and *WDR59*.

**Table 3:** Gene ontology (GO) functional enrichment analysis for the differentially expressed genes for hyperactivity at day 0 in Landrace

Category	Term	Description	*P*-value	DE counts	Total in category
CC	GO:0070062	extracellular exosome	6.58E-06	409	1371
CC	GO:0005737	cytoplasm	1.67E-05	616	2157
BP	GO:0001701	in utero embryonic development	2.73E-05	52	123
MF	GO:0045296	cadherin binding	3.68E-05	68	174
MF	GO:0000166	nucleotide binding	5.37E-05	190	591
CC	GO:0005925	focal adhesion	6.80E-05	79	213
CC	GO:0061700	GATOR2 complex	1.17E-04	8	9
MF	GO:0005524	ATP binding	2.68E-04	242	792
BP	GO:0060048	cardiac muscle contraction	2.89E-04	11	16
CC	GO:0005634	nucleus	2.98E-04	715	2589
CC	GO:0005813	centrosome	7.63E-04	103	310
BP	GO:0010248	establishment or maintenance of transmembrane electrochemical gradient	9.37E-04	5	5

Gene ontology results are presented with category (*CC* cellular component, *BP* biological process, *MF* molecular function), term, description, significant level (*p*-value), gene count in differentially expressed genes (DE) and total number of genes in category

For contrast (iii), seven pathways were over-represented (Table 4), and the most significant pathways include the cellular component “extracellular space” (GO:0005615), “extracellular region” (GO:0005576) and “adenosine catabolic process” (GO:0006154). None of the overrepresented pathways in contrast (i) and (iii) were in common.

**Table 4:** Gene ontology (GO) functional enrichment analysis for the differentially expressed genes for change in hyperactivity between day 0 and 96 h storage in Landrace

Category	Term	Description	*P*-value	DE counts	Total in category
CC	GO:0005615	extracellular space	2.37E-05	12	360
CC	GO:0005576	extracellular region	2.17E-04	9	247
BP	GO:0006154	adenosine catabolic process	2.31E-04	2	3
CC	GO:0009897	external side of plasma membrane	4.33E-04	5	88
MF	GO:0005125	cytokine activity	4.60E-04	3	21
BP	GO:0071985	multivesicular body sorting pathway	7.27E-04	2	6
CC	GO:0032585	multivesicular body membrane	9.55E-04	2	6

Gene ontology results are presented with category (*CC* cellular component, *BP* biological process, *MF* molecular function), term, description, significant level (*p*-value), gene count in differentially expressed genes (DE) and total number of genes in category

### Variant calling

SNP detection in the sequence data of the Landrace animals from contrasts (i) and (iii) identified 103,161 filtered, high-quality variants (Table 5 and Additional file 3). Of these variants, 75% had existing dbSNP entries. The importance of the detected SNPs was investigated by a case-control analysis and by localization in differentially expressed genes.

**Table 5:** Effects of putative SNPs

SNP effect	All dataset	Joined with (i)	Joined with (iii)	Common (i) and (iii)
3’UTR	16,507	1665	72	4
5’UTR	2741	382	16	8
Frameshift	20	2	0	0
Missense	3452	477	27	7
Start lost	8	0	0	0
Stop lost	8	2	0	0
Stop gained	19	1	0	0
Splice region variant	722	90	3	1
Splice acceptor	19	5	0	0
Splice donor	30	6	0	0
Coding sequence variant	4	0	0	0
Inframe deletion	47	1	0	0
Inframe insertion	20	3	0	0
Protein altering variant	1	0	0	0
Synonymous	77,186	704	29	8
**SNP impact**				
High	104	16	0	0
Moderate	3518	481	27	7
Low	8343	761	32	9
Modifier	243,587	22,707	723	323

SNP effect according to Ensembl VEP for putative variants detected using the animals of contrasts (i) and (iii). The results are shown for all filtered variants in the Landrace dataset and variants in differentially expressed genes. Some variants have more than one predicted effect

In search of SNPs that may affect levels of hyperactivity, a case-control analysis was performed between the high and low groups within each contrast. Genotype frequencies of 278 and 19 variants were significantly different between the high and low groups of contrasts (i) and (iii), respectively (Additional file 4). None of these SNPs had a predicted high impact according to Ensembl variant effect predictor (VEP), however, for contrast (i), 18 SNPs had a predicted moderate or low impact, and for all these SNPs the alternate allele was only present in one of the animal groups (high or low sperm hyperactivity). Thirteen of these 18 SNPs were located in neuraminidase 3 (*NEU3*) and one of these, *rs318364485*, was predicted to be deleterious with a SIFT score of 0.05. Further, two of the 18 SNPs were located in chordin like 2 (*CHRDL2*) and hemicentin 1 (*HMCN1*), respectively.

Of the identified 103,161 filtered, high-quality variants, 16 SNPs located in differentially expressed genes of contrast (i) were predicted to have a high impact (Ensembl VEP) (Table 5 and Additional file 5). These 16 SNPs were located in 15 different genes: cilia and flagella associated protein 65 (*CFAP65*), peptidylprolyl isomerase G (*PPIG*), gametogenetin binding protein 2 (*GGNBP2*), protein arginine methyltransferase 2 (*PRMT2*), calcium binding protein 39 (*CAB39*), RuvB like AAA ATPase 1 (*RUVBL1*), coiled-coil domain containing 187 (*CCDC187*), NUT midline carcinoma family member 1 (*NUTM1*), Rap associating with DIL domain (*RADIL*), angiogenic factor with G-patch and FHA domains 1 (*AGGF1*), ribonucleoprotein PTB binding 1 (*RAVER1*), ribosomal protein S9 (*RPS9*) and three uncharacterized genes. Of the 16 high impact SNPs, six had existing rs-IDs, and of the ten remaining, eight could be verified in an independent whole-genome sequencing dataset from the same Landrace population [13]. The SNPs in *CAB39* and *AGGF1* could not be verified in this dataset. When the detected 103,161 filtered, high-quality variants were compared to the results of contrast (iii), no high impact SNPs were localized in the differentially expressed genes.

## Discussion

Transcriptome sequencing was conducted to elucidate genes and pathways associated with sperm hyperactive motility in two pig breeds, Landrace and Duroc. Hyperactive motility of sperm cells is important for fertilization of an oocyte; however, if acquired too early, the sperm cells drain their energy and risk not reaching the oocyte. In this study, we therefore included two contrasts in Landrace: (i) high versus low levels of sperm hyperactivity at the day of ejaculation (day 0) and (iii) high versus low change in levels of sperm hyperactivity between day 0 and 96 h storage. In Norway, porcine liquid diluted semen is recommended to be used within 96 h after collection, hence this storage limit of contrast (iii). We also tried to include a third contrast, day of ejaculation (day 0) in Duroc (ii), however, this was not successful and is discussed in the section “Breed differences” below. Generally, it is of interest to identify putative biomarkers for boar fertility and no studies in the past have looked at testis gene expression and how it is associated with sperm hyperactive motility. Each of the groups within the contrasts consisted of a limited set of animals (*n* = 3–5), which could affect our results by introducing spurious associations. Moreover, individual differences in seminal plasma content was not taken into account in the current study. Seminal plasma is reported to contain decapacitation factors and seems to inhibit the development of hyperactivated spermatozoa [14]. Further, proteins in seminal plasma have been reported to keep the spermatozoa progressively motile, viable and acrosome intact during storage [15]. This means that the results of both the contrast at the day of ejaculation and the contrast looking at difference in hyperactivity after storage could be affected by individual variation in seminal plasma content.

### Genes important for hyperactivity regardless of storage

Of the 3219 differentially expressed genes in contrast (i) and the 102 differentially expressed genes in contrast (iii), 41 were in common, suggesting that these genes are important for sperm hyperactive motility both initially and after storage. The most up-regulated gene in the high hyperactivity group of both contrasts was *DNLZ*, suggesting an important function in sperm hyperactivity in Landrace. *DNLZ*, also known as *HEP* and *HEP1*, is a zinc finger protein that is known to be important for regulation of *HSPA9* ATPase activity in human by enhancing the rate of ATP hydrolysis [16, 17]. HSPA9 seems to be implicated in the maturity and fertilizing ability of spermatozoa [18] and was also differentially expressed in a proteomic study for human sperm motility [19]. Whether an increased HSPA9 ATPase activity by *DNLZ* is the link to sperm hyperactive motility needs to be further investigated.

Adenosine deaminases *ADA* and *ADA2* were differentially expressed in contrasts (i) and (iii), and the gene ontology term “adenosine catabolic process” was significant for contrast (iii). Previous studies have shown that adenosine enhances sperm capacitation and motility through ADA receptors [20–22]. This is interesting as capacitation is necessary for hyperactivation of sperm cells and could explain our results. Other genes in common for contrasts (i) and (iii) that have previously been associated to sperm motility are huntington interacting protein 1 gene (*HIP1*), protamines *PRM1* and *PRM3*, transmembrane protein 225B (*TMEM225B*) and fascin-3 (*FSCN3*). CASA analysis of *HIP1* knockout mice has previously shown reduced velocity, amplitude of lateral head displacement and numbers and percentages of motile, rapid and progressive sperm cells [23]. The study of Khatchadourian et al. [23] suggested that HIP1 was involved in sperm motility and morphology by stabilizing actin and microtubules. *PRM1* and *PRM3* are protamines, which are known for their effect on tight chromatin packaging in sperm cells and association with sperm DNA fragmentation [24]. Sperm motility has also been indirectly associated with protamine through sperm chromatin structure assay parameters [25] and *PRM3* deficient mice was shown to exhibit reduced sperm motility [26]. *TMEM225* has been shown to regulate protein phosphatase 1γ2, which is necessary for sperm capacitation and motility in mice [27], whereas *FSCN3* has been associated with capacitation and was proposed a role in rapid cytoskeletal changes [28]. Although none of these genes have previously been associated to hyperactivity, they have been associated to motility. It is not examined or known which type of motility, and our results suggest that the regulation of these genes are affected in the hyperactive fraction of the sperm.

### Initial levels of hyperactivity

The most significant differentially expressed gene in contrast (i), high versus low levels of sperm hyperactivity at the day of ejaculation, was *ARFGAP1*. The ARFGAP1 protein interacts with ARF1 to regulate membrane traffic between the Golgi and endoplasmic reticulum [29]. *ARFGAP1* is suggested to play a role in the physiological function of sperm because of its high expression in testis [29], and another ARF GAP member, *SMAP2*, has been found important for acrosome formation during spermiogenesis in mice [30]. The results of our study suggest that *ARFGAP1* may play a role for the initial levels of hyperactive motility in ejaculates. Whereas *DNLZ* (discussed above) was the most up-regulated gene in the high hyperactivity group, the novel *ENSSSCG00000037477* was the most down-regulated. According to NCBI, this gene is tetratricopeptide repeat protein 37-like (*TTC37*), which is involved in protein-protein interactions and has mainly been studied in relation with human diseases [31]. This gene has also been associated with fertility in bulls where it was highly expressed in embryos conceived with semen from low fertility sires [32]. The mechanisms behind this gene’s involvement in hyperactive motility and fertility is, however, not known.

The testing for over-represented gene ontology pathways showed that a large proportion of the differentially expressed genes from contrast (i) belonged to the term “extracellular exosome”. The exosome is a vesicle that is released into the extracellular region, and previous studies have found that epididymal extracellular microvesicles are involved in vertebrate sperm maturation (reviewed by [33]). Moreover, boar seminal plasma exosomes have been associated with sperm motility [34]. Differentially expressed genes from this pathway includes fibronectin 1 (*FN1*), which has previously been found correlated with midpiece and tail sperm defects in boars [35], and which has also been correlated to total sperm motility in human [36]. FN1 was also differentially expressed at the protein level in boar seminal plasma between ejaculates with good and poor freezability [37]. Other differentially expressed genes from “extracellular exosome” include members of the heat shock protein family (Hsp70) member 4 (*HSPA4*) and family D (Hsp60) member 1 (*HSPD1*). *HSPA4* is involved in spermatogenesis and a previous study found that mice lacking this gene had a reduced number of spermatozoa and reduced sperm motility [38]. *HSPD1* is shown to undergo tyrosine phosphorylation during capacitation in mouse sperm [39] and it is involved in spermatogenesis in human [40]. *HSPD1* has, however, not been found associated with sperm motility or hyperactive motility in human [41], and its function in boar sperm hyperactivity needs to be further investigated. In addition to *HSPA4* and *HSPD1*, a heat shock protein regulator also belonging to “extracellular exosome”, bcl2 associated athanogene 6 (*BAG6*), was differentially expressed. *BAG6* is the key regulator of heat shock protein *HSPA2*, which is involved in spermatogenesis [42–44] and associated with sperm motility [45]. Other differentially expressed genes of this pathway include quiescin sulfhydryl oxidase 1 (*QSOX1*), angiotensin I converting enzyme (*ACE*), polycystin 1 transient receptor potential channel interacting (*PKD1*), and CD81 molecule (*CD81*). QSOX1 has been suggested a role in sperm cell development in mice, based on immunohistochemical association with the sperm acrosome during maturation [46]. Testicular *ACE* has been found important for sperm motility and male fertility [47] and one of its products, angiotensin II, has been shown to affect sperm motility parameters such as VCL [48], which is one of the CASA parameters describing levels of hyperactivity. *PKD1* is involved in testis development [49, 50] and is known to cause low sperm quality in men with autosomal dominant polycystic kidney disease [51]. Lack of one homozygous genotype of a *PKD1* mutation in stallions also showed its importance for fertility [52], and it has been proposed that *PKD1* might interact with CatSpers, which regulate sperm motility (reviewed by [53]). *CD81* has previously been found expressed in mouse and bull sperm [54] and shown to be involved in spermatogenesis [55], moreover, it has been implied that a reduced fertility of *CD81* knockout mice oocytes is due to failed acrosome reaction in sperm cells [56].

In contrast (i), all genes of the gene ontology term “establishment or maintenance of transmembrane electrochemical gradient” were differentially expressed: *ATP1A1*, *ATP1A2*, *ATP1B2*, *ATP4A* and *BAX*. None of these genes were significantly differentially expressed in contrast (ii), suggesting an importance for initial levels of hyperactivity at ejaculation. *ATP1A1*, *ATP1A2* and *ATP1B2* belongs to the Na,K-ATPases, which use ATP as energy to make an electrochemical gradient across cell membranes. *ATP1A1* has previously been found associated with capacitation in bovine [57], however no studies have indicated such a function of *ATP1A2* and *ATP1B2*. Several studies have however shown that another Na,K-ATPase family member, *ATP1A4*, is associated with sperm motility, capacitation and hyperactivity through keeping an uneven transmembrane distribution of Na^+^ and K^+^ [58–60]. Our study suggest a similar role for the differentially expressed Na,K-ATPase genes in hyperactivity. BAX is a member of the B-cell lymphoma 2 family and affects cell apoptosis [61], which plays an important role during the development of mature spermatozoa [62]. *BAX* deficient male mice have been shown to display increased apoptosis and infertility [63, 64]. Further, *BAX* has previously been associated with sperm motility in human [65]. The mechanism linking *BAX* to sperm motility was not suggested, however, it has been shown that apoptosis initiated during spermatogenesis contribute to poor sperm quality, including sperm motility [66].

Eight of the nine genes in “GATOR2 complex” were differentially expressed. GATOR2 regulates mTOR (mechanistic target of rapamycin) signaling by interacting with Rag GTPases. Even though mTOR has previously been found important for spermatogenesis in rats [67], a similar role of the individual differentially expressed genes of this pathway (*CASTOR1*, *MIOS*, *SEC13*, *SESN1*, *SESN2*, *SZT2*, *WDR24* and *WDR59*) has not previously been shown.

Comparison of our results with a high-throughput proteomic study conducted for human sperm motility [19], showed that 57 out of the differentially expressed genes in contrast (i) were the same as their differentially expressed proteins [See Additional File 6]. These genes were to a large degree related to metabolic pathways such as carbohydrate derivate metabolism, lipid metabolic process and protein metabolic process. Moreover, some of the genes has ATP/ADP/GTP/GDP binding activity or ATPase activity: ADP-ribosylation factor-like protein 8B (*ARL8B*), cytochrome b5 reductase 3 (*CYB5R3*), eukaryotic translation elongation factor 2 (*EEF2*), H1 histone family member N testis specific (*H1FNT*), heat shock protein family A Hsp70 member 2 (*HSPA2*), 60 kDa heat shock protein mitochondrial (*HSPD1*), lonprotease homolog mitochondrial (*LONP1*) and valine-tRNA ligase mitochondrial precursor (*VARS*). These results are not surprising as sperm cells need energy for being motile, as was also concluded by Amaral et al. [19]. Tektin 5 (*TEKT5*) was also in common for contrast (i) and the study of Amaral et al. [19]. This gene belongs to pathways such as sperm flagellum and motile cilium, critical pathways for sperm hyperactivity, and has previously also been associated with sperm motility in mouse [68].

Peroxiredoxin-5 mitochondrial (*PRDX5*), acrosin-binding protein (*ACRBP*) and apolipoprotein A-I (*APOA1*) were differentially expressed in contrast (i). This is supported by a previous study examining capacitation related proteins in boar spermatozoa [10]. It was suggested that PRDX5 plays an important role in regulating energy production in the sperm cell and in sperm-oocyte binding, whereas ACRBP regulates release of acrosin from the acrosome, a proteinase involved in sperm penetration of zona pellucida. *APOA1* has previously been found to induce cholesterol efflux in spermatozoa, a process that regulates motility, hyperactivation and capacitation [69, 70].

### Levels of hyperactivity after storage

The most significant differentially expressed gene in contrast (iii), high versus low change in levels of sperm hyperactivity between day 0 and 96 h storage, was *SLC40A1*. This gene is involved in iron transport, and previous studies have shown that testicular iron deficiency reduce levels of spermatozoa (reviewed by [71]). The exact function for *SLC40A1* in the resulting levels of sperm hyperactivity is not clear and needs to be further investigated. The most up-regulated gene in the high hyperactivity group was *DNLZ*, whereas the most down-regulated gene was *CADPS*. *CADPS* is involved in vesicle exocytosis of various compounds like insulin and neurotransmitters [72] and is tightly coupled to ATP activation and Ca^2+^ influx [73]. The protein has been found mainly expressed and studied in brain cells of human, mice and rats (e.g. [74, 75]), however it is also expressed in other mice tissues [75], and in pigs, it has been suggested as a candidate gene for body weight [76]. A function in sperm hyperactive motility have not previously been implied, however, it could be explained by its role in ATP activation and Ca^2+^-influx.

The two most differentially expressed terms of the gene ontology analysis for contrast (iii) were “extracellular space” and “extracellular region”. In gene ontology analyses, extracellular region is a parent term of extracellular space, and these are the cell regions where junctions between germ cells and Sertoli cells are located [77]. As germ cells mature and migrate through the testis, a cascade of signal transduction events at the junctions are involved, requiring factors such as cytokines, proteases, protease inhibitors, protein kinases, protein phosphatases, GTPases and junctional complex and extracellular matrix proteins (reviewed by [77]). We have previously shown that numerous genes in this part of spermatogenesis are important for different levels of sperm DNA fragmentation [78]. Differentially expressed genes in the current study belonging to the above-mentioned pathways include *ADA2* (already discussed above), and C-X-C motif chemokine ligand 10 (*CXCL10*), TNF superfamily member 10 (*TNFSF10*) and WNT family member 5A (*WNT5A*) (last three genes discussed under “cytokine activity” below). Moreover, phospholipase A2 group IIA (*PLA2G2A)* was differentially expressed, a gene whose protein has previously been associated to sperm motility and fertility in human [19, 79]. Phospholipase A2 is a calcium dependent enzyme which is involved in phospholipid metabolism in membranes and it has been proposed involved in sperm motility also in other species such as sea urchin [80] and fowl [81]. In boars, it has been shown that phospholipase A2 is important for capacitation [82], the process where hyperactive motility is achieved, and the results of this study show that the phospholipase A2 *PLA2G2A* gene is down-regulated in samples with high hyperactivity after storage.

Another significantly overrepresented gene ontology terms for contrast (iii) was “cytokine activity”, which includes the three differentially expressed genes *CXCL10, TNFSF10* and *WNT5A*. Levels of different cytokines have previously been associated to sperm motility and progressive motility in human (e.g. [83, 84]), and our results suggest that cytokines have an effect on hyperactive motility after storage of sperm cells. Cytokines are important for cellular immune responses and are involved in spermatogenesis in the testis by affecting Sertoli – germ cell interactions [77]. *CXCL10* was down-regulated in the group with high change in levels of hyperactivity after 96 h storage. It has previously been shown to cause germ cell apoptosis in mice, where *CXCL10* knock-out mice had reduced apoptosis [85]. Further, *CXCL10* was related to sperm survival in chicken [86]. A role for this gene in sperm hyperactive motility has not previously been indicated, however, previous experiments showed a negative correlation between proportion of apoptotic cells, where apoptosis is initiated during spermatogenesis, and sperm motility in ejaculates [66]. *TNFSF10* is an important signaling molecule for proper germ cell apoptosis as knock-out mice showed a dramatically reduced production of mature spermatozoa [87]. Moreover, *TNFSF10* was found up-regulated in Landrace boars with high levels of sperm DNA fragmentation in our previous study [78], suggesting that this gene affects several sperm quality parameters. Finally, *WNT5A* is involved in self-renewal of spermatogonial stem cells [88], a process which is essential for spermatogenesis.

### Breed differences

In this study, Landrace and Duroc samples were analyzed using the same CASA settings, however, after RNA sequencing, the extreme samples from Duroc did not cluster according to their hyperactive phenotype. The Duroc boars had a higher variability in their hyperactivity levels (Table 1) than the Landrace boars and both the high and low group of Duroc boars had higher levels of hyperactivity compared to Landrace high and low groups. The lack of clustering could suggest that their hyperactivity levels might not be extreme enough, but it could also imply that the settings of the CASA were not appropriate for this breed. For CASA measurements, various instrument settings for different species are most commonly used. For example, the instrument defines and classifies sperm motility patterns differently, and motility characterizations are not identical for different species [89]. It has also been shown previously that different cattle breeds should have different CASA settings due to their large variability in sperm variables [90]. The results of the current study might suggest that breed specific characteristics exists for pigs as well. Our previous study found differences between Landrace and Duroc with regards to hyperactive motility [6]. Landrace sperm cells increased hyperactive motility with 6.3% from day of collection to 96 h storage. Duroc, on the other hand, showed higher initial sperm hyperactivity at day of collection and no significant increase after 4 days of storage. Differences in sperm physiology and semen plasma composition for these two breeds have also been found in other studies [91–93]. The results from the current study suggest that breed specific CASA thresholds should be tested.

### Variant calling

The variant calling was done on a limited set of animals, however, a case-control study pointed towards SNP variants that are present in only one of the sample groups and hence associated to sperm hyperactivity. For contrast (i), 278 SNPs were different between the high and low hyperactivity groups. Eighteen of these variants had a moderate or low predicted impact according to Ensemble VEP and were located in genes *NEU3*, *CHRDL2*, *HMCN1* and uncharacterized genes *ENSSSCG00000002261* and *ENSSSCG00000026662*. *NEU3* is a sialidase whose activity has previously been found necessary for capacitation and zona pellucida binding [94]. It is therefore interesting that 13 of the 18 SNPs with low/moderate impact are located in *NEU3*, including *rs318364485* with a predicted deleterious SIFT score (0.05). Moreover, 90 of the modifier impact SNPs are also located in *NEU3*. The effect should be examined in a larger animal material to see if this SNP could function as a biomarker for sperm hyperactivity.

As this material offers a limited number of animals, additional SNPs could be informative if tested in a larger population. We therefore had a look at SNPs present in the differentially expressed genes. SNPs with a predicted high impact were identified in 15 of the differentially expressed genes of contrast (i). Of these, *CFAP65* has previously been associated with spermatogenesis and flagellar development [95], and loss of function of the chicken ortholog of *CFAP65* has been shown to cause defective sperm motility [96]. Two of the putative high impact SNPs could not be verified in an independent sequencing dataset from the same Landrace population, suggesting that these variants might be false positives or very rare. Even though the other genes with high impact SNPs have not previously been associated with sperm quality, their significant differential expression as well as high impact SNPs make them candidates for further testing as fertility biomarkers.

For contrast (iii), 19 SNPs were different in the case-control analysis between the high and low change in hyperactivity level difference between day 0 and 96 h storage. All of these SNPs were modifier impact, meaning that they are not predicted to have a large impact, however, regulatory SNPs might be located in regions such as 3’UTR [97], which are classified as modifier by VEP, so the identified SNPs could affect levels of hyperactivity after storage through such mechanisms.

## Conclusions

In this study, we performed transcriptome profiling of boar testis tissue from Landrace and Duroc boars based on their levels of sperm hyperactive motility at the day of ejaculation (day 0) and on their change in hyperactive motility between day 0 and 96 h storage. We identified differentially expressed genes and pathways from Landrace boars with high and low levels of hyperactivity on the day of ejaculation (contrast i) and boars with high and low change in hyperactivity between day 0 and 96 h liquid storage at 18 °C (contrast iii). Each contrast consisted of three to five animals per group and the animals showed consistently high or low levels of hyperactivity. Overall, our data suggests that *DNLZ* is important for sperm hyperactivity regardless of storage, whereas *ARFGAP1* and *SLC40A1* were the most significant genes for contrasts (i) and (iii), respectively. More than 100,000 filtered high-quality SNPs were identified in the dataset, and a predicted deleterious SNP in the differentially expressed gene *NEU3* was detected by case-control analysis. The limited number of animals (*n* = 3–5 for each group) could affect the results of this study. The results of this study provide novel insight regarding testis gene expression and its importance for sperm hyperactive motility. Genes and variants identified might be candidate markers useful for predicting boar fertility.

## Methods

### Aim and design of the study

The aim of this study was to identify differential expression of genes in testis tissue from boars with high and low levels of sperm hyperactive motility at ejaculation and change in hyperactive motility after storage. To facilitate this, testis tissue samples were collected at slaughter from AI boars, whose ejaculates had been used for hyperactivity measurements. Samples from boars with repeated extreme high or low sperm hyperactive motility at the day of ejaculation (day 0) and high or low change in hyperactive motility between day 0 and 96 h storage were selected for transcriptome sequencing.

### Materials

Semen collected from 103 purebred Norwegian Landrace boars (*n* = 239 ejaculates) and 88 purebred Duroc boars (*n* = 179 ejaculates) at the AI station run by Norsvin at Hamar, Norway, were the basis for this study. All boars were routinely used for AI and the ejaculates were part of routine collections for the breeding program. The boars were housed in individual 6 m^2^ pens, fed a standard commercial diet and had access to straw and sawdust as rooting materials. The ejaculate samples were collected between February 21st, 2014 and March 20th, 2015 and the age of the boars at semen collection for sampling ranged from 241 to 1041 days (median age = 338 days). Testis tissue samples were collected after slaughter and immediately frozen in liquid nitrogen, before storage at − 80 °C until RNA extraction. For Landrace boars with extreme levels of hyperactivity on day 0, 4 boars with high values and 4 boars with low values were selected (contrast (i)). For Duroc boars with extreme levels of hyperactivity on day 0, 3 boars with high levels and 4 boars with low levels were selected (contrast (ii)). For Landrace boars with high versus low changes in levels of hyperactivity between day 0 and 96 h storage, 5 boars with high levels and 4 boars with low levels were included in the study (contrast (iii)).

### Hyperactivity measurements

The sperm-rich fraction of the ejaculates was collected using the gloved hand technique, as described by Althouse et al. [98]. At the AI station, motility and morphology were subjectively evaluated using phase contrast microscopy (Leica DM 4000B, Leica Microsystems, Wetzlar, Germany) at 37 °C, and ejaculates with < 70% motile and/or > 20% morphologically abnormal spermatozoa were discarded. Ejaculates approved by the quality check were diluted to achieve a concentration of 25 × 10^6^ cells/mL in Androstar® Plus extender (Minitube, 84,184 Tiefenbach, Germany), transferred to airtight tubes containing doses of 89 mL, and stored in liquid conditions at 18 °C until shipment. Only semen accepted for AI was used in this study. During the 15 min long transportation from the AI station to the laboratory, the samples were packed in a styrofoam box to ensure a stable temperature. At the laboratory, semen was transferred to 15 mL falcon tubes and the samples were taken for CASA analysis at the day of collection (day 0) and after storage at 18 °C for 96 h (day 4), as this is the time limit recommended by Norsvin for using the dose. In order to be included in the selection of extreme animals, the boars needed at least three consistent measurements on % hyperactivity (Table 1).

### RNA extraction and sequencing

The RNeasy Midi Kit (Qiagen) was used to extract total RNA from testis tissue. RNA concentrations were measured using a NanoDrop ND-1000 Spectrophotometer (NanoDrop Technologies, DE, USA) and the quality was examined by the 28S:18S rRNA ratio using the RNA 6000 Nano LabChip® Kit on 2100 Bioanalyzer (Agilent Technologies, CA, USA). All samples showed RNA integrity numbers (RIN) > 7.8 and a 260/280 ratio > 1.9. The sequencing was conducted by the Norwegian Sequencing Centre at the Centre for Ecological and Evolutionary Synthesis, University of Oslo (http://www.sequencing.uio.no). Libraries were prepared using TruSeq mRNA stranded HT kit (Illumina) on a Sciclone NGSx liquid automation system (Perkin Elmer). Final library quality check was performed on Fragment Analyser (Advanced Analytical Technologies, Inc) and by qPCR (Kapa Biosciences). Libraries were sequenced on an Illumina HiSeq 4000 according to manufacturer’s instructions. Image analysis and base calling were performed using Illumina’s RTA software v2.7.7. The reads were 100 base library pair single reads and filtered to remove those with low base call quality using Illumina’s default chastity criteria. All samples were run over two sequencing lanes and the number of reads per sample ranged from 65 to 196 million with an average of 98 million reads. The data has been deposited in NCBI’s Gene Expression Omnibus (GEO) [99] with GEO series accession number GSE141541.

### Processing and differential expression of RNA sequencing data

Trimmomatic v.0.36 [11] was used to trim reads by removing leading bases with Phred_33_ quality scores < 5, trailing bases with Phred_33_ quality scores < 3, using a sliding window of 4 bases and removing the 5′ terminal base if the average Phred_33_ score of the 4 bases was < 15, and completely discard trimmed reads with less than 36 remaining bases. The high quality reads were subsequently mapped to the pig genome *Sscrofa* build 11.1 using the Star software v.2.5.2a and default parameters while simultaneously adding unique read groups to the files [100]. Gene prediction coordinates (release 11.1.90) were obtained from Ensembl (http://www.ensembl.org). Samtools v.1.3.1 [101] was used to merge bams from the same sample and to convert bam files to sam files before HTSeq [102] was run with the reverse stranded option to calculate the number of reads mapped to each gene. The differential expression analysis was conducted using the package edgeR v.3.12.1 in the R environment [103]. The two breeds and different contrasts were analyzed separately, and the samples were divided into a high-low hyperactivity level contrast based on their hyperactivity values. Filtering was done to keep only genes that achieved at least one count per million in at least half of the samples, and the data was normalized for differences in the abundance of read counts mapped to genes between samples using the TMM (trimmed mean of M-values) normalization method [104]. This adjusts any differential expression analysis for varying sequencing depths and different library sizes and is part of the basic modeling procedure in edgeR. Variance in gene expression was estimated using a tagwise dispersion model before differential expression was detected with a likelihood ratio test model (option glmFit in edgeR). FDR was calculated using the Bejamini-Hochberg algorithm [105], and an FDR < 0.05 was considered significant. Gene ontology analyses were run using the R package “goseq” v.1.26.0 [106] using BioMart as implemented in R (biomaRt v.2.30.9) [107] to incorporate pig IDs and the Wallenius approximation method to account for length biases. Pathways with an overrepresented *p*-value < 0.001 was considered significant.

### Variant calling

Preprocessing of bam files was performed using Picard (sorting, duplicate marking, and indexing) [108]. Variant calling on the Landrace RNA sequencing data was conducted using Samtools v.1.3.1 mpileup and bcftools call [101]. Detected variants were filtered, using bcftools filter and vcftools [109], on alternate allele count (> 2), QUAL (> 25), mapping quality (> 10) and read depth (> 10). Furthermore, both reference and alternate allele had to be present on both strands, and a threshold of at least 4 bp to the next SNP and 10 bp to the next indel was also applied. The filtered variants were subsequently annotated using the Ensembl Variant Effect Predictor (VEP) [110] to classify variants and their impact. SnpSift v.4.3 was subsequently used to estimate case-control (i.e. high versus low hyperactivity) using an allelic model (CC_ALL) and Fisher exact test [111]. Variants were considered significantly different at *p* < 0.001. SNP validation was done in silico by matching detected variants to known pig entries collected using VEP and by comparison to an existing independent whole-genome sequence dataset of related boars [78].

## Supplementary information

**Additional file 1.** Differentially expressed genes for hyperactivity at ejaculation (day 0) in Landrace. The results are presented with Ensembl gene id, gene symbol, gene name, fold change and significance level (FDR).

**Additional file 2.** Differentially expressed genes for change in levels of hyperactivity between day 0 and 96 h storage in Landrace. The results are presented with Ensembl gene id, gene symbol, gene name, fold change and significance level (FDR).

**Additional file 3.** Variants identified in the Landrace animals of contrasts (i) and (iii). The variants are presented per chromosome with Ensembl VEP information.

**Additional file 4 **Variant case-control analysis. Chromosome, position, number of homozygous, heterozygous and total variants in cases and controls, Fisher exact test *p*-value for the case control allelic model according to SnpSift (CC_ALL, see Methods), and contrast for significance (Table X1). Consequence, impact, gene id, ensembl gene id, existing variation and SIFT scores are included in Table X2.

**Additional file 5.** Variants occuring in differentially expressed genes. Variants in genes identified for hyperactivity at Day 0 in Landrace (contrast (i), Table X1), change in levels of hyperactivity between day 0 and 96 h storage in Landrace (contrast (iii), Table X2), and genes in common for contrasts (i) and (iii) (Table X3). The SNPs are presented with Ensembl gene id, gene name, significance level of differentially expressed gene (FDR), chromosome (SSC) and position, effect, impact, existing ID and SIFT score.

**Additional file 6.** Overlapping results of contrast (i) and proteins involved in human sperm motility. Comparison of our results with the study of Amaral et al. [19] showed that 57 out of the differentially expressed genes in contrast (i) were the same as their differentially expressed proteins.

## Data Availability

The datasets generated and analyzed during the current study are available in the additional files and in NCBI’s GEO database with accession number GSE141541.

## Abbreviations

ACE: Angiotensin I converting enzyme
ACRBP: Acrosin-binding protein
ADA: Adenosine deaminase
ADA2: Adenosine deaminase 2
ADP: Adenosine diphosphate
AGGF1: Angiogenic factor with G-patch and FHA domains 1
AI: Artificial insemination
ALH: Amplitude of lateral head displacement
APOA1: Apolipoprotein A-I
ARFGAP1: ADP ribosylation factor GTPase activating protein 1
ARL8B: ADP-ribosylation factor-like protein 8B
ATP: Adenosine triphosphate
ATP1A1: ATPase Na+/K+ transporting subunit alpha 1
ATP1A2: ATPase Na+/K+ transporting subunit alpha 2
ATP1A4: ATPase Na+/K+ transporting subunit alpha 4
ATP1B2: ATPase Na+/K+ transporting subunit beta 2
ATP4A: ATPase H+/K+ transporting subunit alpha
BAG6: bcl2 associated athanogene 6
BAX: BLC2 associated X, apoptosis regulator
Bp: Base pairs
CAB39: Calcium binding protein 39
CADPS: Calcium dependent secretion activator
CASA: Computer-assisted sperm analysis
CASTOR1: Cytosolic arginine sensor for mTORC1 subunit 1
CatSper: Cation channel of sperm
CCDC187: Coiled-coil domain containing 187
CD81: CD81 molecule
CFAP65: Cilia and flagella associated protein 65
CHRDL2: Chordin like 2
CXCL10: C-X-C motif chemokine ligand 10
CYB5R3: Cytochrome b5 reductase 3
DNLZ: DNL-type zinc finger
EEF2: Eukaryotic translation elongation factor 2
FDR: False discovery rate
FN1: Fibronectin 1
FSCN3: Fascin-3
GDP: Guanosine diphosphate
GEO: Gene Expression Omnibus
GGNBP2: Gametogenetin binding protein 2
GTP: Guanosine triphosphate
H1FNT: H1 histone family member N testis specific
HIP1: Huntington interacting protein 1 gene
HMCN1: Hemicentin 1
Hsp70: Heat shock protein 70 kilodalton
HSPA2: Heat shock protein family A Hsp70 member 2
HSPA4: Heat shock protein family A (Hsp70) member 4
HSPA9: Heat shock protein family A Hsp70 member 9
HSPD1: 60 kDa heat shock protein mitochondrial
LIN: Inearity
LONP1: Lonprotease homolog mitochondrial
MIOS: MEIOSIS regulator for oocyte development
mTOR: Mechanistic target of rapamycin
NEU3: Neuraminidase 3
NUTM1: NUT midline carcinoma family member 1
PPIG: Peptidylprolyl isomerase G
PKD1: Polycystin 1 transient receptor potential channel interacting
PLA2G2A: Phospholipase A2 group IIA
PRDX5: Peroxiredoxin-5 mitochondrial
PRM1: Protamine 1
PRM3: Protamine 3
PRMT2: Protein arginine methyltransferase 2
QSOX1: Quiescin sulfhydryl oxidase 1
RADIL: Rap associating with DIL domain
RAVER1: Ribonucleoprotein PTB binding 1
RIN: RNA integrity number
RNA: Ribonucleic acid
RPS9: Ribosomal protein S9
RUVBL1: RuvB like AAA ATPase 1
SD: Standard deviation
SEC13: SEC13 homolog nuclear pore and COPII coat complex component
SESN1: Sestrin 1
SESN2: Sestrin 2
SLC40A1: Solute carrier family 40 member 1
SMAP2: Small arfGAP2
SZT2: KICSTOR complex subunit
TEKT5: Tektin 5
TMEM225B: Transmembrane protein 225B
TMM: Trimmed mean of M-values
TNFSF10: TNF superfamily member 10
TTC37: Tetratricopeptide repeat protein 37-like
UTR: Untranslated region
VARS: Valine-tRNA ligase mitochondrial precursor
VEP: Ensembl variant effect predictor
VCL: Curvilinear velocity
WDR24: WD repeat domain 24
WDR59: WD repeat domain 59
WNT5A: WNT family member 5A
WOB: Wobble
